# Characteristics of Retractions from Korean Medical Journals in the KoreaMed Database: A Bibliometric Analysis

**DOI:** 10.1371/journal.pone.0163588

**Published:** 2016-10-05

**Authors:** Sun Huh, Soo Young Kim, Hye-Min Cho

**Affiliations:** 1 Department of Parasitology, College of Medicine, Hallym University, Chuncheon, Korea; 2 Department of Family Medicine, Gangdong Sacred Heart Hospital, College of Medicine, Hallym University, Seoul, Korea; 3 Infolumi Co., Seongnam, Korea; Peking University First Hospital, CHINA

## Abstract

**Background:**

Flawed or misleading articles may be retracted because of either honest scientific errors or scientific misconduct. This study explored the characteristics of retractions in medical journals published in Korea through the KoreaMed database.

**Methods:**

We retrieved retraction articles indexed in the KoreaMed database from January 1990 to January 2016. Three authors each reviewed the details of the retractions including the reason for retraction, adherence to retraction guidelines, and appropriateness of retraction. Points of disagreement were reconciled by discussion among the three.

**Results:**

Out of 217,839 articles in KoreaMed published from 1990 to January 2016, the publication type of 111 articles was retraction (0.051%). Of the 111 articles (addressing the retraction of 114 papers), 58.8% were issued by the authors, 17.5% were jointly issued (author, editor, and publisher), 15.8% came from editors, and 4.4% were dispatched by institutions; in 5.3% of the instances, the issuer was unstated. The reasons for retraction included duplicate publication (57.0%), plagiarism (8.8%), scientific error (4.4%), author dispute (3.5%), and other (5.3%); the reasons were unstated or unclear in 20.2%. The degree of adherence to COPE’s retraction guidelines varied (79.8%–100%), and some retractions were inappropriate by COPE standards. These were categorized as follows: retraction of the first published article in the case of duplicate publication (69.2%), authorship dispute (15.4%), errata (7.7%), and other (7.7%).

**Conclusion:**

The major reason for retraction in Korean medical journals is duplicate publication. Some retractions resulted from overreaction by the editors. Therefore, editors of Korean medical journals should take careful note of the COPE retraction guidelines and should undergo training on appropriate retraction practices.

## Introduction

Retraction can be defined as “the ‘removal’ from the literature of a paper determined to be sufficiently fraudulent, falsified, mistaken or not reproducible that the authors or editors act to acknowledge its invalidity in the public record” [1]. Retraction has also been described as “a mechanism for correcting the literature and alerting readers to publications that contain such seriously flawed or erroneous data” [2]. The major reason for retraction was once the occurrence of an honest scientific error [3], but according to recent research, the number of retractions has increased rapidly [4–6]. The Committee on Publication Ethics (COPE) has published “Retraction Guidelines” to encourage uniform and comprehensive practices in issuing retractions [2]. In the guidelines, there are recommendation about the purpose, form, reasons, and timing of retractions [2]. However, it is not known whether retraction issuers have adhered to the guidelines. Nor is it known whether the reason for the retraction and compliance with the retraction guidelines differs according to the country in which the journal publishing the retraction is based. Characteristics of retraction have not been investigated at the country level. Identifying the characteristics of retraction at the single country level is facilitated by the fact that abstracts of all medical journals based in Korea have been indexed in the KoreaMed database (http://koreamed.org/).

This study aimed to investigate the characteristics of retraction in Korean medical journals in the KoreaMed database. Specifically, chronological frequencies of retraction, issuer, reason for retraction, compliance with retraction guidelines, and appropriateness of retraction were determined.

## Materials and Methods

The retraction articles evaluated in this study were located in KoreaMed on January 28, 2016 and selected by a co-author of this paper (HMC), who has 30 years of experience as a medical librarian. The search query was “retraction of publication” [PT] OR “retraction” [TI]. PT is an abbreviation of publication type and TI is that of article title. The full text of articles from the search results were checked on the journal homepage. Articles that contained “retraction” as a medical term were excluded and only retraction articles as a publication type were included. Three reviewers (SH, SYK, HMC) each examined all of the retraction notices independently and determined the characteristics of retraction. The three came to a consensus over the characteristics through a group discussion. The characteristics assessed were as follows:

- Content related to the retraction: date of retraction, interval from publication to retraction.
- The issuer: Subjects who issued retractions were classified as editor, author, other, unknown, and joint
- Reason for retraction: The reason was classified as fabrication, falsification, plagiarism, duplicate publication, authorship, scientific error, other ethical issue, and unknown.
- Compliance with 2009 COPE Retraction Guidelines: Seven items were determined according to the guidelines as described in Table 1.
- Appropriateness of retraction: Following the 2009 COPE guidelines, inappropriate retractions were classified as follows [2]: retraction of the first article published in a case of duplicate publication, retraction that could have been resolved as an erratum retraction due to an authorship dispute, or other inappropriate retraction.

**Table 1:** COPE retraction notice recommendations and criteria used for review.

Recommendation	Interpreted as adherence to the recommendations
1. Be linked to the retracted article wherever possible (i.e. in all electronic versions)	The retracted articles could be linked to the journal website.
2. Clearly identify the retracted article (e.g. by including the title and authors in the retraction heading)	Clearly stated the retracted article in the retraction notice PDF file
3. Be clearly identified as a retraction (i.e. distinct from other types of correction or comment)	Clearly stated as retraction in the retraction notice PDF file
4. Be freely available to all readers	The retraction notice can be read without logging in to the journal website.
5. State who is retracting the article	Clearly stated who was retracting the article.
6. State the reason(s) for retraction (to distinguish misconduct from honest error)	Clearly stated the reason(s) for retraction in the notice.
7. Avoid statements that are potentially defamatory or libelous	No potentially defamatory or libelous statement in the retraction notice.

## Results

Out of 217,839 articles in KoreaMed published from 1990 to January 2016, the publication type of 111 articles was retraction (0.051%). Among them, one retraction article retracted four papers; therefore, the total number of retracted papers was 114.

**Fig 1:**
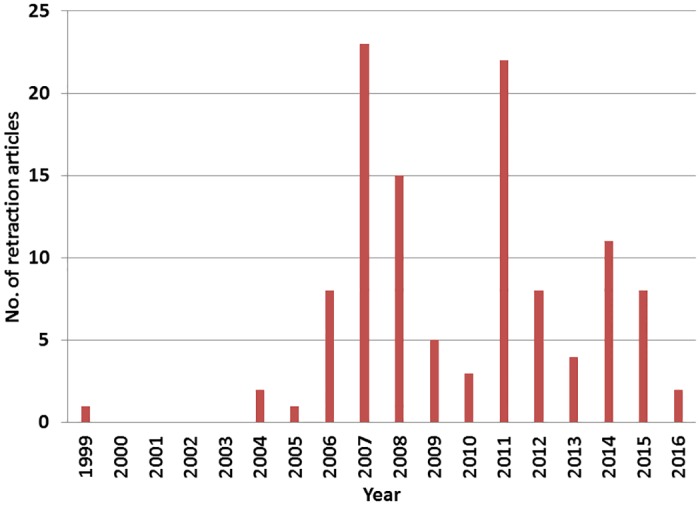
Number of retractions listed in the KoreaMed database from 1999 to 2016.

Interval from publication to retraction: The average interval was 45.9 months (range, 1–225 months). From 1990 to 2009, the average was 34.7 months (range, 2–128 months), while from 2010 to March 2016, it was 55.9 months (range, 1–225 months). Annual trends: A retraction article first appeared in the KoreaMed database in 1999. The most frequent publication of retraction articles occurred in 2006 (Fig 1). There was no notable trend in frequency from 1999 to 2016.

Issuer: Sixty-seven retractions were issued by the author (58.8%); 20 (17.5%) were issued jointly by the author, journal editor, and publisher; 16 (14.0%) by the editor; 3 (2.6%) by the committee for publication ethics of the publishing society; two (1.8%) by the institution of author affiliation; and in six cases (5.3%), no source information was provided

Reasons for retraction (Table 2): The most common reasons for retraction were duplicate publication (66 cases, 57.9%), plagiarism (10, 8.8%), scientific error (5, 4.4%), and authorship dispute (4, 3.5%). There were 4 other reasons: Institutional Review Board problem, conflict of interest-related problem, printing company error, and copyright problem. There was no traceable description of the reason for retraction in 23 cases (20.2%).

**Table 2:** Reasons for retraction (n = 114).

Reasons	Frequency (%)
Duplicate publication	66 (57.9)
Plagiarism	10 (8.8)
Scientific mistake	5 (4.4)
Author dispute	4 (3.5)
Others	4 (3.5)
Unknown	23 (20.2)

Compliance with the 2009 COPE retraction guidelines: Compliance surpassed 80% on six out of seven items in the guidelines., ‘state the reason (s) for retraction’, achieved compliance in 79.8% of the articles All of the retraction articles addressed two items: ‘be linked to the retracted article wherever possible’ and ‘clearly identify the retracted article’ (Table 3).

**Table 3:** Adherence of retraction notices listed in KoreaMed to the Committee on Publication Ethics (COPE) guidelines (n = 114).

Requirement	No. adhering (%)
Be linked to the retracted article wherever possible (i.e., in all electronic versions)	114 (100.0)
Clearly identify the retracted article (e.g., by including the title and authors in the retraction heading)	114 (100.0)
Be clearly identified as a retraction (i.e., distinct from other types of correction or comment)	113 (99.1)
Be freely available to all readers^a^	101 (88.6)
State who is retracting the article	108 (94.7)
State the reason(s) for retraction (to distinguish misconduct from honest error)	91 (79.8)
Avoid statements that are potentially defamatory or libelous	113 (99.1)

^a^Lack of availability included articles for which content was not available from the journal web site due to the lack of a journal homepage at the time of retraction.

Appropriateness of retraction according to COPE retraction guidelines: Retraction was appropriate in 50 cases (43.9%). While 26 retractions (22.8%) were inappropriate, in 38 cases (33.3%), appropriateness could not be determined. Out of 26 inappropriate cases, the reasons were as follows (Table 4): retraction of the first article published in duplicate publication (18 cases, 69.2%), authorship dispute (4, 15.4%), what could have been corrected by an erratum (2, 7.7%), partial retraction (1), and a duplicate publication in which both articles were retracted (1).

**Table 4:** Reasons for inappropriate retraction (n = 26).

Reasons	Frequency (%)
Retraction of the first published articles in duplicate publication	18 (69.2)
Author dispute	4 (15.4)
Correctable by erratum	2 (7.7)
Partial retraction	1 (3.8)
Retraction of both articles in duplicate publications	1 (3.8)

## Discussion

The data we collected showed certain differences from results previously gathered from PubMed, such as the chronological trend and interval between publication and retraction. According to the results from PubMed, the number of retractions increased year by year [4,5]; however, in Korea, there has been no such trend. Our results showed that there were two peaks in 2007 and 2011, when the publication of retraction articles was concentrated in a small number of journals. Because duplicate publication was drawing a great deal of attention in Korean medical societies at that time, duplicate publications were retracted frequently.

In the present study, it was found that retractions were most often issued by the author (58.8%) or jointly by the author, publisher, and editor (15.8%); therefore, the author was involved in 74.6% of retractions. This result was similar to the 64% noted by Wager and Williams [5] and 81% by Budd et al. [7].

The reasons for the114 retractions in the KoreaMed database differed from previous reports. The most remarkable difference was the high proportion of duplicate publication (57.8%). The reason may be due to several duplication publication scandals and a publication ethics awareness campaign in Korea [8]. In other reports, duplicate publication as a reason for retraction comprised 17% of 312 MEDLINE retractions from 1988 to 2008 [5] and 15.8% of 742 PubMed retractions from 2000 to 2010 [4]. Another difference was that the reason for the retraction was unknown in 20.2% of the cases in the KoreaMed data. On the other hand, unidentified reasons comprised only 5% of the data in Wager and Williams [5], 8.2% in Steen [4], and 1.5% of 235 retractions from Medline from 1966 through August 1997 [7]. Our findings indicate that when retractions are issued for journals published in Korea, the author or editor should take care to clearly describe the reason for the retraction.

In the evaluation of reasons for retraction, the differences in the proportions among these studies could have depended on the definition of misconduct and honest scientific errors used in each case. If misconduct was limited to fabrication and falsification, the proportion of errors was greater than that of misconduct [4,5]. If misconduct was expanded to include duplicate publication and plagiarism, the proportion of misconduct was greater than that of errors [6].

The range of misconduct according to the Korean government is similar to that reported by Fang et al. [6]. According to the "Regulation on the management of national research and development" published by the Korean government, research misconduct includes fabrication, falsification, authorship dispute, plagiarism, and other inappropriate research activities (http://www.law.go.kr/LSW/lsInfoP.do?lsiSeq=142802#0000). In the present study, the proportion of misconduct was 81 out of 114 (74.6%), which was greater than the 67.4% of Fang et al. [6]. Interestingly, there was no fabrication or falsification in Korea. This may be explained by the fact that it is nearly impossible to identify fabrication or falsification unless an internal whistleblower has raised the issue. Another possible explanation is that no researcher had published fabricated or falsified data in journals based in Korea.

No study has previously evaluated whether retraction notices have complied with COPE retraction guidelines [2]. In the present study, retraction notices in the KoreaMed database complied well with each item in the COPE retraction guidelines. Relatively lower compliance items were as follows: be freely available to all readers (88.6%); state who is retracting the article (94.7%); and state the reason(s) for retraction (79.8%). Unavailability to all readers does not refer to subscription-based access in the cases we found since there are no subscription-based articles in the KoreaMed database, but rather that the retraction article was not available in full text in the journal home page.

Appropriateness of retraction was determined by the COPE retraction guidelines. The following were the types of inappropriate retraction: retraction of the first article published in duplicate publication; author dispute; an error that could have been corrected by an erratum; partial retraction; and both articles retracted in duplicate publication. It is not possible to compare the present results with other studies since there is no literature on the appropriateness of retractions according to COPE retraction guidelines. Out of 114 retractions, 51 (44.7%) were appropriate retractions, 26 (22.8%) were inappropriate retraction, and in 37 cases (32.5%), appropriateness could not be determined. Out of 38 of the undetermined cases, 18 cases involved duplicate publication in which it was unclear which article was published earlier. The COPE retraction guidelines recommend against partial retraction; however, it is one of the publication types in the MEDLINE database [9]. This topic deserves further discussion by professionals in publication ethics and library science.

The above results have demonstrated how the characteristics of retraction articles from the KoreaMed database differ from the MEDLINE/PubMed database described in other reports as follows: a higher proportion of duplicate publication as the reason for retraction; no fabrication or falsification; the appearance of retractions not complying with COPE retraction guidelines such as retraction of the first article published in duplicate publication; different proportions of issuer types; a greater proportion of retractions for an undetermined reason; and overreactions to errors in which a retraction was issued instead of an erratum.

It is necessary to train journal editors on how retraction articles should be written. They should understand that “applied and executed appropriately, retractions are a sign that a journal takes its responsibilities to publication integrity seriously and should never be considered a sign of failure” [10]. At the same time, not only the appropriate writing of retraction articles but also the effort to prevent retractions should be included in editor training programs. To prevent duplicate publication, checking each submitted manuscript with CrossCheck, a similarity checking program for scholarly journal editors, should be a mandatory task of the editorial office or editorial board members [11,12].

There were limitations to this study. First, the classification of retraction notices involved some subjectivity, although the three authors reviewed the data independently and came to an agreement after discussion. Second, the search of the KoreaMed database may have missed some retraction notices if they had been misclassified into another publication type and the title did not include “retraction” or if there was no English citation data. Thus the retraction articles may have been undercounted if some retractions were not issued correctly.

In conclusion, the most common reason for retractions published in Korean medical journals is duplicate publication. Some retractions appeared to have resulted from an overreaction on the part of the journal editors. Therefore, training on appropriate retraction practices is needed for editors of Korean medical journals, and the editors should be exhorted to adhere to the retraction guidelines more meticulously. Similar studies on the retraction practices of journal editors in other countries would be welcome, allowing for international comparison of training needs and possible collaboration on training efforts.

## Supporting Information

S1 Table Detailed characteristics of retractions from Korean medical journals indexed in the KoreaMed Database. (XLSX)
